# Exploitation of *E. coli* for the production of penicillin G amidase: a tool for the synthesis of semisynthetic β-lactam antibiotics

**DOI:** 10.1186/s43141-021-00263-7

**Published:** 2021-10-15

**Authors:** Krishika Sambyal, Rahul Vikram Singh

**Affiliations:** 1 University Institute of Biotechnology, Chandigarh University, Gharuan, Punjab India; 2 Academy of Scientific and Innovative Research (AcSIR), Ghaziabad, 201002 India

**Keywords:** 6-Aminopenicillanic acid, Antibiotics, *E. coli*, Penicillin G amidases/acylases, Recombinant

## Abstract

**Background:**

Penicillin G amidase/acylases from microbial sources is a unique enzyme that belongs to the N-terminal nucleophilic hydrolase structural superfamily. It catalyzes the selective hydrolysis of side chain amide/acyl bond of penicillins and cephalosporins whereas the labile amide/acyl bond in the β-lactam ring remains intact.

**Main body of abstract:**

This review summarizes the production aspects of PGA from various microbial sources at optimized conditions. The minimal yield from wild strains has been extensively improved using varying strain improvement techniques like recombination and mutagenesis; further applied for the subsequent synthesis of 6-aminopenicillanic acid, which is an intermediate molecule for synthesis of a wide range of novel β-lactam antibiotics. Immobilization of PGA has also been attempted to enhance the durability of enzyme for the industrial purposes.

**Short conclusion:**

The present review provides an emphasis on exploitation of *E. coli* to enhance the microbial production of PGA. The latest achievements in the production of recombinant enzymes have also been discussed. Besides *E. coli*, other potent microbial strains with PGA activity must be explored to enhance the yields.

**Graphical abstract:**

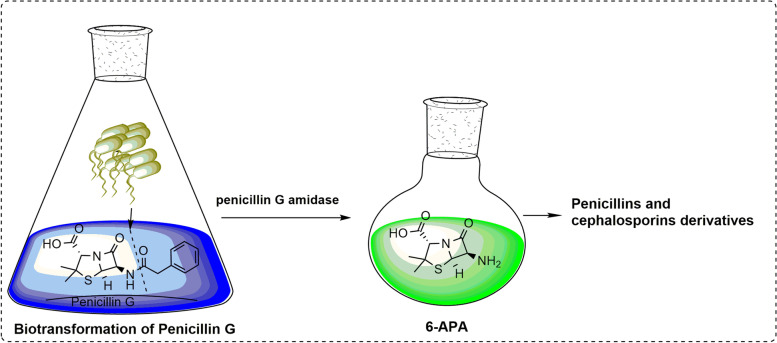

## Background

**Fig. 1:**
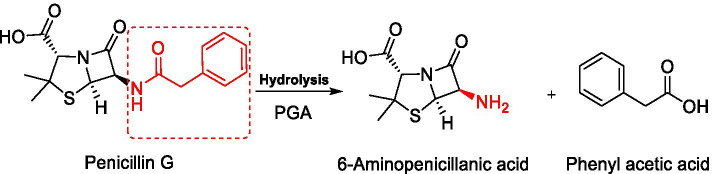
Hydrolysis of penicillin G by PGA to synthesize 6-APA

Penicillin G amidases or acylases (PGA) (penicillin amidohydrolase: EC 3.5.1.11) is one of the principal enzymes at an industrial level used to catalyze the enzymatic hydrolysis of various penicillins by cleaving their amide bond to yield 6-APA and its corresponding organic acid [1]. Penicillin G (PG, also known as benzyl penicillin), penicillin V (PV, also known as phenoxymethylpenicillins), and other bulk penicillins are the substrates which are chemically or enzymatically transformed for synthesis of wide range of novel β-lactam antibiotics such as amoxicillin, ampicillin, and cephalosporins [2]. Due to several disadvantages of chemical synthesis, currently much preference is given to enzymatic hydrolysis than the chemical methods as it is more specific, cheaper, and occurs at milder conditions [3]. The conversion of penicillin G to 6-aminopenicillanic acid (6-APA) and phenyl acetic acid (PAA) occurs via hydrolysis of the amide bond in its side chain catalyzed by PGA under slight alkaline pH resulting in the transfer of phenyl acetyl moiety from 6-APA to water [4] (Fig. 1). PGA is the second most commercially used enzyme worldwide, followed by glucose isomerase [5] and penicillin G was the first β-lactam antibiotic discovered in 1940s which is primarily active against gram-positive bacteria [4]. PGA is widely distributed among microbes like bacteria, fungi, yeasts, and actinomycetes [6], which are often cultivated at temperatures lower than 30 °C [2]. 6-APA further facilitates the route to synthesize a variety of semisynthetic antibiotics with improved antibacterial and pharmacological characteristics [7]. Due to the enormous use of antibiotics, pathogens have developed resistance against them with time. Thus, to overcome this concern, the only method is to synthesize new semisynthetic antibiotics [8]. The recent efforts for the overproduction of PGA are achieved through genetic engineering by constructing novel recombinant host/vector systems [7]. Besides this, the fermentation operations are run by quantitatively analyzing the kinetics to achieve optimum conditions for PGA production.

To make the process economic and commercial at industrial scale, PGA is immobilized on a matrix by various means like membrane or fiber entrapment, adsorption, and covalent binding to allow re-utilization as well as enhancing the enzymatic conversion of penicillins under acidic or alkaline conditions [9, 10]. The first executed hydrolysis by immobilized PGA involved penicillin G and cephalosporin G as substrates [11]. This method was quickly implemented to be used by the pharmaceutical industry since the mild conditions were appropriate for the stability and activity of PGA [12]. *E. coli* PGAs generally have a heterodimeric structure as in *E. coli* ATCC 11105 PGA which is synthesized as a single cytoplasmic precursor with 23-kDa α-subunit and 65-kDa β-subunit [13], maybe due to the unusual protein formation mechanism [14]. The mature *E. coli* PGA is located in the periplasm [2] which allows a rather simple extraction procedure as only cell permeabilization is required in such case [15]. PGAs belong to the N-terminal nucleophilic hydrolase structural superfamily with a catalytic nucleophile (Ser, Cys, or Thr) as the active site for cleaving an amide bond at the N-terminus [16]. PGAs are potentially useful biocatalysts in various ways such as protection of amino and hydroxyl groups during peptide synthesis and resolution of racemic mixtures of chiral compounds [17, 18]. They are also known to work as a promising candidate for linker cleavage studies of structures for combinatorial chemistry due to their high substrate specificity concerning the acyl residue [19].

## Main text

### Discovery of penicillin G amidase

Enzymes are the ideal biocatalysts which provide eco-friendly environmental conditions for different processes to occur mildly, thus of great importance to the industries [5]. PGA was firstly reported in the mycelium of *Penicillium chrysogenum* Q176 in the year 1950 as an enzyme with potential to catalyze the hydrolysis of penicillin G into PAA and an unknown compound called “penicillin” (6-APA) in the form of hygroscopic crystalline needles giving 158–159 °C melting point [20, 21]. There are various microbial strains of bacteria, fungi, yeasts, and actinomycetes like *Escherichia coli*, *Pseudomonas melanogenum*, *Bacillus megaterium*, *Streptomyces lavendulae*, *Achromobacter* sp., *Bovista plumbea*, *Kluyvera* sp., *Penicillium chrysogenum*, *Actinoplanes* sp., *Proteus rettgeri*, and *Mucor griseocyanus* [3] available which produce PGA, i.e., exhibit PGA activity either in their natural wild types or after recombinant processing. But the most efficient and highly preferred enzymes are derived from *E. coli* and *Bacillus megaterium* strains [22]. In 1960, the hydrolysis of penicillin G using PGA from *E. coli* was reported by Bayer and Beecham while Kaufmann and Bauer reported the hydrolysis of penicillin to 6-APA [7]. Due to the ability of *E. coli* PGA to hydrolyze a wide variety of phenyl acetyl substituted compounds, it has been widely used in various formulations [23]. The production kinetics of some PGA producing microorganisms has been presented in the Tables 1 and 2.

**Table 1:** Production kinetics of PGA producing wild type strains

Microbes	Media	pH	Temp. (°C)	Incubation (h)	Reference
*Penicillium chrysogenum* Q176	–	7.6–8.0	35–38		[20]
*Escherichia coli* ATCC 9637	K_2_HPO_4_, K_2_HPO_4_, (NH_4_)_2_SO_4_, MgSO_4_.7H_2_0, phenylacetic acid, sodium glutamate	7.0	30	8-10	[24]
*E. coli* ATCC 11105 (Wild)	Liquid corn-steep liquor, peptone, and glucose medium	7.0	26	18	[21, 25]
*Bacillus* sp. PGS10	K_2_HPO_4_, MgSO_4_.7H_2_O, CaCl_2_.2H_2_O, PAA, tryptone, yeast extract, sucrose	7.0	28	16	[26]
*Aspergillus fumigatus*	Skim milk with phenylglycine methyl ester as inducer	7.5–8.5	30	144	[27]
*Mucor griseocyanus*	Skim milk with phenylglycine methyl ester as inducer	7.0–8.0	30	144	[27]
*Alcaligenes sp.* (purified PGA)	Peptone, beef extract, PAA medium	8.0	28	18	[28]
*Bacillus megaterium* (Wild)	Amino acid and cheese whey medium	7.0–8.0	30	24	[5]
*Achromobacter xylosoxidans* subsp. *indiges* subsp. nov.	Peptone, beef extract, PAA medium		28		[29]
*B. megaterium*	Carboxyl methyl cellulose medium	7.0	60	120	[30]
*Mucor griseocyanus*	Czapek liquid medium: NaNO_3_, KHPO_4_, MgSO_4_·7H_2_O, KCl, lactose	6.5	30	120	[31]

**Table 2:** Production kinetics of PGA producing recombinant strains

Microbes	Host system	Media	pH	Temp. (°C)	Incubation (h)	Reference
*Arthrobacter viscosus*	*E. coli*	M9 medium with succinate	6.0–8.0	28	48–150	[32]
*Providencia rettgeri*	*Saccharomyces cerevisiae*	YPGal, YPLac, YPD media	6.2	30	72–120	[33]
*E. coli* ATCC11105	Recombinant *E. coli χ*6212/pRT4	Luria-Bertani (LB) media	7.0	28	48	[34]
*E. coli*	*E. coli* MC1000	M9 medium with IPTG inducer		28		[35]
*P. rettgeri*	*Pichia pastoris*	Yeast, peptone, dextrose (YPD) medium	6.0	30	24–144	[36]
*A. faecalis* ATCC 1908	*E. coli* BL21(DE3)	Minimal M9 medium with glucose		28	6	[37]
	Recombinant *E. coli* BL21(DE3)	LB medium and M9 minimal medium	7.4	28	24	[38]
*A. faecalis* ATCC 19018	*E. coli* JM109	M9 and LB medium with rhamnose inducer	7.0	37		[19]
*Kluyvera citrophila*	*E. coli* BL21 (DE3)	TB medium (Tatof-Hobbs)	7.28	28	12	[39]
*-*	Recombinant *E. coli*	Minimum medium with cheese whey powder	7.0	29	24	[40]
*-*	Ultraviolet induced mutated wild *E. coli* strains BDCS-N-S21, NW50 and N-FMu10	LB broth	7.0	37	12	[41]
*B. badius*	*E. coli* DH5*α*	Yeast, fructose and mineral components		28	24	[42]
*Thermus thermophilus (Tth)* HB27	*E. coli*	LB media with CaCl_2_	4.0–5.0	75	9.2 h half life	[43]
*–*	Engineered *E. coli*	Yeast extract, tryptone, chemicals, and casamino acid medium	7.0	28	16	[44]
*E. coli* ATCC 11105 (Wild)	*E. coli* JM109 (Recombinant)	Casein medium supplemented with ampicillin and IPTG (0.1 mM)		26		[17]
*Achromobacter* sp. CCM 4824	*Pichia pastoris* X-33	Yeast extract, peptone, glycerol, methanol	7.2	28	24	[45]
*Providencia rettgeri* (PrPGA)	*E. coli* BL21 (DE3)	LB medium, kanamycin, IPTG	7.0	37	12–24	[46]
*Alcaligenes faecalis* (AfPGA)	*E. coli* BL21 (DE3)	LB medium, kanamycin, IPTG	7.0	37	12–24	[46]
*Achromobacter xylosoxidans* (AxPGA)	*E. coli* BL21 (DE3)	LB medium, kanamycin, IPTG	7.0	37	12–24	[46]
*Achromobacter* sp.	*P. pastoris* X33 pENS2	mineral medium with biotin, glycerol	5.5	30	–	[47]

### Exploitation of *E. coli* for penicillin G amidase production

*E. coli* has been exploited in every way possible, but still it shows to be the best among others in terms of PGA production. The microbial production of PGA, either in its native strain (Table 1) or recombinant hosts (Table 2), has been extensively studied, especially for PGA from *E. coli* (EcPGA) [2]*.* PGA is an intracellular enzyme and its production is achieved by growing distinct strains in standard mediums containing carbon, nitrogen sources along with an inducer (mostly, PAA) in appropriate concentrations. The study of substrates, nutrient composition, type of cultivation, bioreactors, etc., plays a key role in optimizing conditions for a biocatalyst to grow and minimize the production of toxic substances. Various purification schemes have been applied in order to get a partially purified or purified PGA enzyme, like ammonium sulfate precipitation followed by desalting which in turn is used as a biocatalyst to produce 6-APA after hydrolyzing penicillins for synthesizing antibiotics [48]. Partially purified PGA can be immobilized for industrial applications without additional chromatographic purification [2]. Chemical deacylation can also be performed for 6-APA production, but it is non-specific, environmentally hazardous, and expensive; thus, it is not preferred much [48]. The designing of industrial plant for large scale production must be done using improvised engineering approaches with highly robust techniques like strain manipulation, cultivation methods, and downstream processing [49]. The overexpression in recombinant *E. coli* hosts for the industrial usage can be succeeded by optimization of the operational strategies [50].

### Production of recombinant strains and PGA purification

**Fig. 2:**
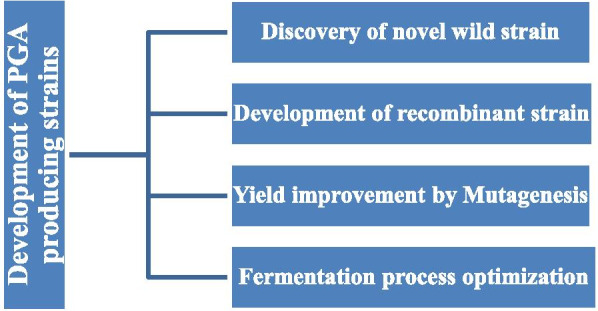
Strategies adopted by researchers for the production of PGA

The vigorous growth characteristics of *E. coli* along with its conventional metabolism and physiology make it a simplistic host system. The EcPGA serves as an excellent enzyme for industrial uses but the promoter of its endogenous *pac* gene is weak and, thus, unsuitable for large scale production. Hence, its manipulation can be done to produce a recombinant PGA (Fig. 2) by overexpressing the native gene in high-copy episomal plasmids to build up gene dosage so as to boost all expression steps under regulation of a strong promoter-operator system, like transcription, translation, translocation, periplasmic processing, and folding. The augmentation of translation can be done by increasing the stability of *pac* mRNA, modifying the region of ribosome binding site, etc., leading to a mature PGA giving higher levels of activity [15, 51]. The *pac* genes from bacterial strains other than *E. coli* have also been heterologously expressed in *E. coli* like genes from *Arthrobacter viscosus* [52], *B. megaterium* [53], *Achromobacter xylosoxidans* [54], *P. rettgeri* [55], *A. faecalis* [56], *K. cryocrescens* [57], and *Thermus thermophilus* [43]. Their PGAs can surpass EcPGA when expressed in *E. coli* in terms of particular enzymatic properties like wide operation range, molecular stability, and environmental tolerance [2]. An overproduction of mature PGA has been performed by cloning *pac* gene from *E. coli* ATCC 11105 into pUC 9 rather than pBR 322. The emerged vector (*E. coli* pUPA-9) when transformed into *E. coli* 5 K resulted in production of 10 mg PGA/L of cells due to high gene copy number. Hydrophobic interaction chromatography and anion exchange was used to purify the enzyme from periplasmic fraction of *E. coli* pUPA-9 [58]. The successful expression of *pac* genes from *E. coli* and *Providencia rettgeri* in *Saccharomyces cerevisiae* revealed that contrary to bacterial hosts, where PGA is retained in the periplasm; the yeast cells secrete enzyme directly into the medium, and *E. coli pac* is poorly expressed in yeast [33]. A *pac* gene from parental *E. coli* ATCC11105 was cloned and expressed in recombinant *E. coli* χ6212/pRT4 which produced 1000 units PGA/g of cell dry weight that is 23-folds more than the parent strain (43 units PGA/g of cell dry weight). The replacement of original *pac* promoter by strong *ptrc* promoter of the vector pYA292 led to this increase in the yield. Later, 16 units/mg of protein was purified near to homogeneity by hydrophobic interaction chromatography giving 60% recovery of PGA by two-step of purification [34].

However, to enhance PGA production in different recombinant *E. coli* expression hosts and identify the reason behind decrease in post translational yield, PGA from *E. coli* ATCC 11105 was used as a model recombinant protein. It was observed that the main processes limiting enzyme production were intracellular proteolytic degradation of the newly synthesized PGA precursor by reduction of the growth rate of cells, protective fusions, and translocation through the plasma membrane. Therefore, to elevate PGA expression at molecular level in recombinant *E. coli* strains, intracellular proteolysis and translocation were manipulated, and an appropriate host system with suitable cultivation medium was selected. The cultivation of an *E. coli* strain BL21 (DE3), which is a natural deficient of ATP-dependent proteinase, Lon and outer membrane proteinase, OmpT [59], was done in a medium without a proteinaceous substrate that provided an increased PGA yield by 10-folds [38]. It has been studied earlier that PGA yields can be improved by the use of minimal medium with different carbon sources since it is sensitive to complex proteinaceous substrates [60]. By employing the rhamnose inducible expression system in *E. coli*, production of recombinant PGA from *A. faecalis* ATCC19018 was carried out. The rhamnose inducible promoter, also serving as a carbon source, was used to control the cloning of desired gene into a multi copy vector. A PGA activity of 4500 U/L was achieved with 0.96 U/mg of specific activity in the cell free extract [19]. Production of PGA from *Kluyvera citrophila* was optimized in *E. coli* BL21 (DE3), and effects of physical parameters such as temperature and pH were investigated. The cells reached their stationary phase after 24 h of growth, and yield was increased by more than 2.4-fold in TB medium. Culture performance was studied, and 9600 U/L activity and 24.4 cell density were achieved [39]. The use of cheese whey as an inducer and carbon source has been done to produce PGA by recombinant *E. coli* W3110/pPA102*.* A specific activity of 781 U/g was attained at use of 5 g/L of cheese whey and 3% dissolved oxygen which shows that it can be successfully used as an inducer and carbon source for PGA production using constructions driven by the lac promoter [40]. *E. coli* HB101 and JM109 are among the host systems which perform well for the production of PGA [61]. A *pac* gene with its promoter was obtained by using SSP-PCR and direct genome sequencing from *Bacillus badius* and expressed in different *E. coli* hosts. A recombinant PGA was obtained in *E. coli* DH5α with maximum 1820 U/L activity. Ni-NTA chromatography was used to purify the PGA which showed stability over a wide pH range of 6.0–8.5 with a maximum activity at pH 7.0 and activity on a wide β-lactam substrate range. The *k*_*cat*_*/Km* values revealed that enzyme had strongest preference for penicillin G (1025.0 mM/s) followed by cephalothin, cephalexin, and ampicillin [62]. Screening for PGA producing *E. coli* isolates was carried out to study cloning and recombinant expression. A positive clone gene was cloned in plasmid pGEM-T easy vector, and BL21 host cells were explored for high PGA levels. The comparative study of wild and recombinant strain revealed that in *E. coli* BL21, inducer IPTG (1 mM) can increase the level of PGA to 150 U/g (wet weight) of recombinant bacteria, i.e., three times more than PGA activity of wild type *E. coli* strain [63]. A gene homologous to *pac* from *E. coli* was isolated from the outer side of cytoplasmic membrane of *Thermus thermophilus* (Tth) HB27. Its overexpression was difficult in mesophilic host due to complex maturation and nature. Thus, by using chaperone co-expression and calcium supplementation of the culture medium, its PGA was efficiently overexpressed in *E. coli.* Further, affinity chromatography was performed to obtain recombinant PGA by conformation through SDS-PAGE and MALDI-TOF analysis. It was determined for activity and hydrophobic acyl-chain penicillins were preferred as substrates with more specificity towards penicillin K possess highest specificity constant value (16.12 mM/s) while penicillin G had approx. 1.10^−4^ mM/s [43]. But this strain can be engineered by mutagenesis of selected active site residues to hydrolyze penicillin G to a definite extent [23]. For efficacious production, PGA was overexpressed in an engineered *E. coli* and released in medium with low conductivity to allow direct application of the extracellular fraction to the anion-exchange chromatography column. Further, harvested cells were used for purification of enzyme by using strong anion-exchange (Q) column yielding PGA activity of 16.3 U/mg at 871 U/g DCW, i.e., up to 3 folds [44]. Strong cation-exchange membrane adsorbers were used in a one-step purification process to get high productivity of bounded PGA (98%) with a residual enzyme activity of 80–85%. *E. coli* 5KpHM12 real cell lysate was processed under optimal conditions, and PGA was isolated with a purification factor of 101.3 (4.97 U/mg) [64]. Based on the TFF-AMEC, a single-step downstream process has been reported to purify *E. coli* PGA with high yield [49].

### Mutagenesis for strain improvement

**Scheme 1:**
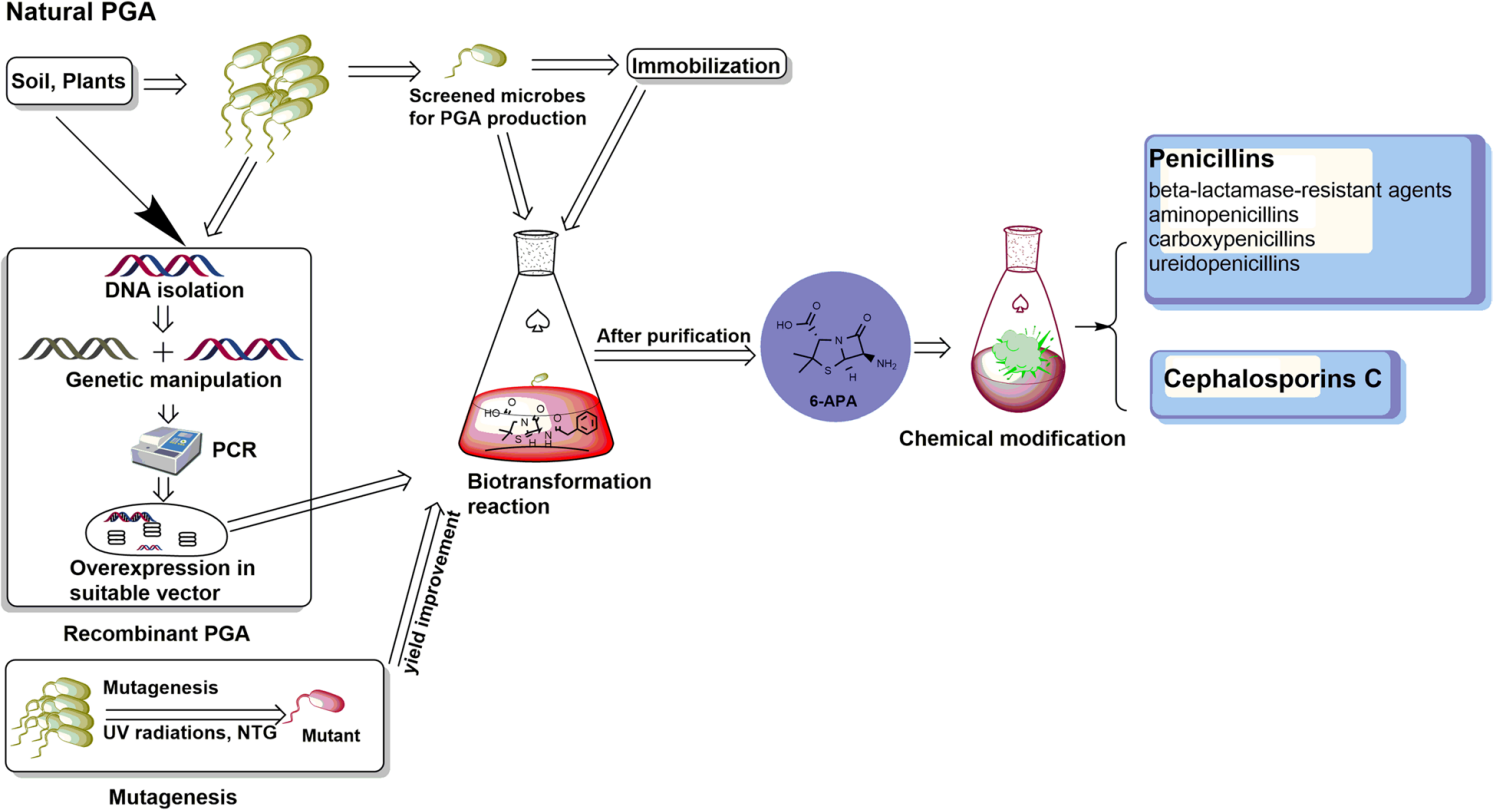
Schematic representation of overall production of PGA, yield improvement strategy and synthesis of 6-APA for the synthesis of β-lactam mediated antibiotics

Mutagenesis is another powerful tool used for strain improvement (Scheme 1) for the production of PGA. Different mutants have been produced by treating *E. coli* with acridine orange yielding high levels of PGA and inactivated β-lactamase [65]. A four times more productive mutant than the parent strain has been produced by chemical mutagenesis of *E. coli* ATCC 11105 with NTG [66]. UV radiations have also been known to improve the catalytic efficiency and substrate specificity of PGA producing bacterial strains [67]. Locally isolated *E. coli* strains were mutated using UV radiation by exposing diluted cultures to UV lamps at varying time and distances. BDCS-N-M74 was the hyper-producing mutant exhibiting 3 fold (22.5 mg of 6-APA/h/mg wet cells) increases in PGA activity as compared to that in the parent strain (6.7 mg of 6-APA/h/mg wet cells) with limited expression of β-lactamase. Due to these mutations, the efficiency for microbes to produce enzymes/metabolites increases whereas higher expose can cause lethal changes in the microbe [41]. ep-PCR is a popular method to upgrade and improvise the enzyme properties by increasing frequency of mismatched incorporation of nucleotides into newly synthesized PCR products [68]. ep-PCR coupled with high-throughput screening assay was applied to *E. coli pac* gene to improve enzyme activity of its PGA. The strain pUC19-*pac*wt11 with 0.24 U/ml of activity was used as a template for the construction of mutants by ep-PCR. The best mutant PA M2234 had a specific activity 4.0 times higher than that of enzyme from wild strain and also displayed higher stability at pH 10. DEAE-Sepharose column was used for purification providing 33-fold with 1.34 U/mg specific activity for wild strain and 43-fold with 5.51 U/mg specific activity for mutant [17].

### Immobilization of penicillin G amidase

The commercialization of an enzyme is dependent upon its durability which directly supports product formation by repeating enzyme-reaction cycles. To reduce the process cost of any molecule at industrial scale, the most important factors are an improved log life in growth curve, enzyme stability, and reusability. For all such purposes, immobilization process uses an inert matrix for surface attachment of enzymes, even at extreme of pH and temperature at exceptionally high substrate concentrations in different solvents [10, 69]. Therefore, enzyme immobilization has gained tremendous attraction among scientific communities. Immobilization of PGA has played an important role to make its processing economically feasible at an industrial level [48]. It is due to the successful development of immobilized PGA that replaced the chemical route for 6-APA production [70]. There are several methods such as adsorption, micro-encapsulation, fiber entrapment, copolymerization, cross-linking, and covalent attachment used for immobilization of PGA to increase its stability, facilitate its recovery, and re-use in many hydrolysis cycles. Different methods have been reviewed by Parmar and co-workers [48]. Silica support, glycoxyl agarose, alumina beads, nylon fibers, ethylene glycol dimethacrylate, zerogel, Eupergit C, and sepa beads are some carriers for liquid PGA immobilization [71]. Another methodology known as CLEA is a combination of immobilization as well as purification without much highly purified enzyme. It performs direct immobilization of an enzyme from crude fermentation broth and the first examples of CLEAs were derived from PGA. Due to the limited thermal stability and a low tolerance of free enzymes to organic solvents, PGA stabilization by immobilization becomes an ideal solution for industries [72]. Varying range of other methods like binding, enzyme crystal or powder packaging, and prefabricated carrier materials have been used to immobilize PGAs from microbes such as *Bacillus megaterium*, *A. faecalis*, and *E. coli* [73]. The cells of *E. coli* and *A. faecalis* have also been permeabilized using gelatin, polyvinyl alcohol, and agar matrices giving satisfactory results [15, 55]. Some immobilized preparations of *E. coli* whole cells and its mutants with PGA activity have been made by entrapping in gluten matrix, open pore gelatin matrix [74], and polymethacrylamide beads [75] proving to be effective in penicillin G hydrolysis. For the hydrolysis as well as synthesis of β-lactam antibiotics, cross-linked enzyme crystals of EcPGA featuring characters of pure enzyme and high organic solvent tolerance are evident to work well [76]. To minimize the decrease in catalytic activity in presence of high concentrations of organic co-solvents, an artificial microenvironment generation around the immobilized enzyme can also be done [77]. Under non-denaturing conditions, CLEAs of EcPGA have been prepared by its physical aggregation and cross-linking with glutaraldehyde [78]. For industrial application of PGA, its covalent binding to commercial epoxy-activated acrylic beads (Eupergit C) has ensured refinement in operational stability [79]. But PGA immobilization on Sepabeads-EP is more stable as compared to Eupergit C [80]. The fabrication of macroporous weak cation-exchange methacrylate polymers was done by Wang et al. [81] and Chen et al. [82] to immobilize PGA using hydrophobic interaction chromatography for concentration and purification of enzyme. Cheng and co-workers [55] used pore matrix cross-linking with glutaraldehyde to immobilize the permeabilized whole-cell PGA from *A. faecalis* enhancing PGA activity by 7.5-folds and yielding 75% 6-APA by bioconversion of penicillin G. By suspension polymerization, magnetic hydroxyl particles have been activated with epoxyl chloropropane and used for PGA immobilization giving constant activity at ∼94% for up to 80 cycles [11, 81]. A covalent immobilized PGA on glutaraldehyde activated NH_2_-PVC membranes has been used for 6-APA production giving high catalytic activity up to 4000 μmol min m^2^ and retaining 45% of activity. The immobilized PGA had *K*_*M*_ value (125.8 mM) 23 times higher than that of the free enzyme (5.4 mM) [83]. The immobilization of PGA on magnetic Fe_2_O_3_/Fe_3_O_4_@SiO_2_-CHO nanocomposites via the Schiff's reaction also showed excellent pH stability, thermal stability, and reusability as compared to free enzyme. Sixty-seven percent of the initial activity was retained even after 12 cycles of enzyme usage [84]. NIPAM (N-isopropylacrylamide) with active ester groups is a thermo-responsive, biocompatible polymer which has also been used for immobilization of PGA and the resulting enzyme–polymer conjugate possessed a close hydrolytic activity to that of the free enzyme. Thus, it is suitable for synthesis of the semi-synthetic cephalosporin and cephalexin which is also an important β-lactam antibiotic, by the reaction of D-phenylglycine amide with 7-ADCA (aminodeacetoxycephalosporanic acid) [85].

### Application of 6-aminopenicillanic acid for the production of β-lactam antibiotics

β-lactam antibiotics are one of the common antibiotics having broad spectrum activity against several Gram-negative and Gram-positive pathogens. For treating various bacterial infections, they have been successfully used as a drug over the past few decades across the globe [86]. For the synthesis of β-lactam antibiotics and their derivatives, 6-APA is an important skeleton molecule (Scheme 1). For the first time in 1959, Batchelor and co-workers discovered 6-APA from fermentation of penicillin. This was an important breakthrough for the discovery of novel semi-synthetic β-lactam antibiotics [87]. Further, scientists attempted for a semi-synthetic route to synthesize other novel antibiotics. The addition of different side chains to 6-APA were also done to generate novel antibiotics with different activity and better pharmacokinetics which served to provide resistance from β-lactamases [88, 89]. The core structure of these antibiotics is 3-carbon and 1-nitrogen ring known as β-lactam ring which is highly reactive. PBP are the enzymes responsible for cross-linkage of peptidoglycan components present in the bacterial cell wall [90]. The mechanism behind the antibiotic effect possessed by β-lactams is that they mimic the structure of natural D-Ala-D-Ala substrate of PBP and inhibit its work. This is due to similarity in the positions of CO-N bond in the β-lactam ring of the penicillin and the CO-N bond in D-alanyl-D-alanine which is the target of transpeptidation. Thus, penicillin binds to the site proposed for D-alanyl-D-alanine and halts the cell wall synthesis causing cell lysis [91, 92], although, due to the increased resistance shown by several bacterial strains towards these antibiotics, their work has been afflicted. These strains show resistance to β-lactams by producing an enzyme known as β-lactamases which cleaves the 4-membered β-lactam ring [93]; therefore, inactivating the drug leading to its inefficiency to bind to the target PBPs. If the antibiotic is unable to bind to the target PBP, it would not be able to hinder in the cell wall synthesis causing no harm to the bacteria. It has been revealed by sequence analysis that PBPs and β-lactamases come from a common ancestor. The first bacterial strain to exhibit resistance to some antibacterial agents like penicillin G, erythromycin, streptomycin, and tetracycline was *Staphylococcus aureus* as observed in the year 1947. Gram-positive microbes (*S. epidermidis*, *S. aureus*, *S. pseudintermedius*, etc.) produce β-lactamases into their external surroundings as exoenzymes, while in gram-negative microbes (*Klebsiella*, *Pasteurella*, *Escherichia*, *Haemophilus*, *Salmonella*, *Pseudomonas* sp., etc.), it is kept back in their periplasmic space [91]. It has been evident that antibiotic-resistance genes could also pass from bacteria to other strains or species to procure additional gene combinations. Methicillin/oxacillin-resistant *Staphylococcus aureus* (MRSA), penicillin-resistant *Streptococcus pneumonia* (PRSP), and vancomycin-resistant *Enterococci* (VRE) are some multiple drug resistant organisms. For the bacterial resistance, medicinal chemistry approach has been applied for the discovery of novel antibiotics [94, 95]. Several natural antibiotic scaffolds were chemically modified to produce the new antibiotics with higher pharmacological activity. The main motive of semi-synthetic synthesis is to resolve the bacterial resistance issue, improve the spectrum, improve the oral absorption, and enhance pharmacokinetics [87, 96].

In 2018, the market size of β-lactams antibiotics and β-lactamase inhibitors was estimated at about $27,126 million and is expected to reach about $34,170 million in 2028 [97]. Market size of the PGA is also increasing with the increasing interest of β-lactam antibiotics in the market. At the international market, few pharmaceutical industries are playing an important role in the production of PGA such as Aumgene Biosciences, Fermenta Biotech Limited, Amicogen, Incorporation, Crawford Wisdom International, LGM Pharma, Hangzhou Biodoor Biotechnology Co. Ltd., Taizhou Doyin, etc., and many others. Due to the increment in drug resistance by pathogens, an emergency for the production of novel antibiotics has been created. These new bacterial infections with high drug resistance are an emerging concern threatening public health and here comes the need of novel antibiotics across the world [92]. Therefore, biochemists are paying more attention in semi-synthesis or synthesizing derivatives of natural drugs [76, 98]. Even though the currently available antibiotics will soon find new drug resistant strains, the present unavailability needs to be satisfied first. Studies on the nature of resistance and other molecular details propose a desire for producing novel agents to overcome bacterial resistance [99].

## Conclusion

PGA-mediated transformation is a green process for the synthesis of semisynthetic β-lactam antibiotics which has significant industrial importance. Gene manipulation by recombinant technology, mutagenesis, and immobilization techniques have played an extraordinary role in commercialization of this technology at industrial scale by contribution towards enhancing its properties providing stable and high yield performance strain for the production of 6-APA. Among all the strains, *E. coli* is the only strain which is extensively exploited for the production of PGA whereas biotechnological application like genetic engineering has played a significant role to overcome the yield limitations of native genes in *E. coli.* Other potent microbial strains with PGA activity must also be worked upon to enhance the yields. Since the bacterial strains have been known to show resistance against the previously used antibiotics, there is a great need for development of novel β-lactam antibiotics soon.

## Data Availability

NA

## Abbreviations

6-APA: 6-Aminopenicillanic acid
CLEA: Cross-linked enzyme aggregates
ep-PCR: Error-prone polymerase chain reaction
IPTG: Isopropyl ß-D-1-thiogalactopyranoside
MALDI-TOF: Matrix-assisted laser desorption/ionization-time of flight
NTG: N-methyl-N-nitro-N-nitrosoguanidine
PAA: Phenyl acetic acid
PBP: Penicillin-binding proteins
PGA: Penicillin G amidase/acylases
SDS-PAGE: Sodium dodecyl sulfate polyacrylamide gel electrophoresis
SSP-PCR: Single specific primer polymerase chain reaction
TFF-AMEC: Tangential flow filtration anion-exchange membrane chromatography
UV: Ultraviolet
